# Integrative analysis of oncogenic fusion genes and their functional impact in colorectal cancer

**DOI:** 10.1038/s41416-018-0153-3

**Published:** 2018-06-29

**Authors:** Yuri Choi, Chae Hwa Kwon, Seon Jin Lee, Joonghoon Park, Jong-Yeon Shin, Do Youn Park

**Affiliations:** 1 Department of Pathology, Pusan National University School of Medicine, and Biomedical Research Institute, Pusan National University Hospital, Gudeok-ro 179, Seo-Gu, Busan, 49241 Republic of Korea; 2 Graduate School of International Agricultural Technology and Institute of Green-Bio Science and Technology, Seoul National University, Pyeongchang-gun, Gangwon-do, 232-916 Republic of Korea; 3 Genomic Medicine Institute (GMI), Medical Research Center, Seoul National University, Seoul, 159-781 Republic of Korea

**Keywords:** Cancer genomics, Colon cancer, Tumour biomarkers, Cancer genomics, Colon cancer

## Abstract

**Background:**

Fusion genes are good candidates of molecular targets for cancer therapy. However, there is insufficient research on the clinical implications and functional characteristics of fusion genes in colorectal cancer (CRC).

**Methods:**

In this study, we analysed RNA sequencing data of CRC patients (147 tumour and 47 matched normal tissues) to identify oncogenic fusion genes and evaluated their role in CRC.

**Results:**

We validated 24 fusion genes, including novel fusions, by three algorithms and Sanger sequencing. Fusions from most patients were mutually exclusive CRC oncogenes and included tumour suppressor gene mutations. Eleven fusion genes from 13 patients (8.8%) were determined as oncogenic fusion genes by analysing their gene expression and function. To investigate their oncogenic impact, we performed proliferation and migration assays of CRC cell lines expressing fusion genes of *GTF3A*-*CDK8*, *NAGLU- IKZF3*, *RNF121- FOLR2*, and *STRN-ALK*. Overexpression of these fusion genes increased cell proliferation except *GTF3A-CDK8*. In addition, overexpression of *NAGLU-IKZF3* enhanced migration of CRC cells. We demonstrated that *NAGLU-IKZF3*, *RNF121-FOLR2*, and *STRN-ALK* had tumourigenic effects in CRC.

**Conclusion:**

In summary, we identified and characterised oncogenic fusion genes and their function in CRC, and implicated *NAGLU-IKZF3* and *RNF121-FOLR2* as novel molecular targets for personalised medicine development.

## Introduction

Colorectal cancer (CRC) is a major cause of cancer morbidity and mortality in the world.^1^ Recently, promising molecularly targeted therapies have been developed, such as cetuximab (Erbitux®; Merck KGaA, Germany), an epidermal growth factor receptor (EGFR)-targeted monoclonal antibody, and bevacizumab (Avastin®; Genentech Inc., USA), a vascular endothelial growth factor (VEGF)-targeted monoclonal antibody, for treatment of metastatic CRC.^2, 3^ However, the clinical benefit of targeted therapy remains limited in CRC. Therefore, discovery and development of new molecular targets for therapy is necessary for the treatment of refractory and metastatic CRC.

Through recent advances of sequencing technology, such as next-generation sequencing (NGS), various driver mutations of colorectal cancer were identified, including adenomatous polyposis coli (*APC*), tumour protein p53 (*TP53*), SMAD family member 4 (*SMAD4*), phosphatidylinositol-4,5-bisphosphate 3-kinase catalytic subunit alpha (*PIK3CA*), KRAS proto-oncogene, GTPase (*KRAS*), AT-rich interaction domain 1A (*ARID1A*), SRY-box 9 (*SOX9*) and family with sequence similarity 123B (*FAM123B*).^4^ Fusion genes are good candidates of molecularly targeted therapy, such as the breakpoint cluster region protein (*BCR*)*-*Abelson murine leukaemia viral oncogene homologue 1 (*ABL*) fusion gene in chronic myeloid leukaemia patients, and the anaplastic lymphoma kinase (*ALK*) fusion gene in non-small cell lung cancer.^5, 6^ There are several reports of fusion genes in CRC, including neuron navigator 2 (*NAV2*)*-*transcription factor like 7 (*TCF7L1*) and R-spondin (*RSPO*) fusion genes.^4, 7^ Furthermore, Kloosterman et al. reported that various oncogenic fusions were identified in 2.5% of colon cancer, including proto-oncogene B-Raf (*BRAF*), neurotrophic receptor tyrosine kinase 3 (*NTRK3)*, proto-oncogene Ret (*RET*), and *RSPO* fusion genes.^8^ However, the clinical implications and functional characteristics of fusion genes in CRC are unclear.

In the present study, we identified 24 fusion genes from 19 patients out of a total of 147 colon cancer patients through next-generation RNA sequencing (RNA-seq). To analyse the importance of these oncogenic fusion genes, we validated in vitro the functions of cyclin dependent kinase 8 (*CDK8*), Ikaros family zinc finger protein 3 (*IKZF3*), folate receptor beta (*FOLR2*), and *ALK* fusion genes in CRC cell lines.

## Materials and methods

### RNA seq and analysis of fusion genes

Paired-end RNA seq was performed in our previous study.^9^ Briefly, fresh frozen tissue samples were collected from patients who had resection of the primary tumour at the Pusan National University Hospital and Chonnam National University Hwasun Hospital from 2008 to 2012. In total, 147 tumour samples, including 47 matched normal samples, were analysed. Tumour samples with at least 60% tumour cells without significant mucin or inflammatory cell contaminations examined by mirror image histological analysis were used in the present study. After total RNA was isolated by using RNAiso Plus (Takara, Japan), RNA-seq libraries were generated by TruSeq RNA Sample Preparation Kit according to the manufacturer’s instructions (Illumina, U.S.). Sequencing data were aligned to the National Center for Biotechnology Information (NCBI) human reference genome (hg19) and also aligned to a custom human reference cDNA.^10^

Fusion genes were filtered by GFP algorithm. We determined 101 in-frame shift fusion genes that were not expressed in normal tissues. We applied additional criteria, such as spanning reads ≥10 and chromosomal distance ≥100 kb, to identify intrachromosomal rearrangements. Cross-validation was performed using deFuse^11^ and FusionMap.^12^

### Non-synonymous somatic mutation analysis

We repeated non-synonymous somatic mutation analysis on 19 CRC patients positive for fusion genes. Single nucleotide variants (SNVs) were determined based on Fisher Strand values >30.0 and Qual By Depth values <2.0 using Genome Analysis Toolkit (GATK, version 2015.1-3.4.0-1-ga5ca3fc). SNVs were filtered according to the following criteria: (1) read depth at position ≥10, (2) alteration read depth ≥2, (3) allele ratio at position ≥3%, (4) region in exon, (5) type of change: frameshift, non-synonymous, stop-gain, or stop-loss. To remove potential germline variants, we used dbSNP137 at minor allele frequency >1% of samples^13^ and variants from four matched normal tissue samples.

### Reverse transcription-polymerase chain reaction and Sanger sequencing

Fusion genes were validated by reverse transcription-polymerase chain reaction (RT-PCR) with primers detecting the fusion gene break sites. Total RNA was extracted from fresh frozen tissues and CRC cell lines by using RNAiso Plus (Takara, Japan). RNA samples (500 ng) were reverse-transcribed into cDNA using M-MLV Reverse Transcriptase (Promega, U.S.). PCR reactions were conducted for 4 min at 94 °C, and 35 cycles for 40 s at 94 °C, 40 s at melting temperature (TM) according to each primer set, 40 s at 72 °C, and 7 min at 72 °C. Detailed information of primers is listed in Table S1. PCR products after PCR purification (Cosmogenetech, Korea) were confirmed by Sanger sequencing. To validate the transfection status of vectors transfected into CRC cell lines, RT-PCR was performed according to the same method as described above (Tables S1 and S2).

Quantitative RT-PCR was performed by using SYBRGreen (Applied Biosystems, Life Technologies, U.S.) and the fluorescence reader, Corbett Rotor-Gene 6000 (Qiagen Inc., U.S.). Data were normalised to glyceraldehyde 3-phosphate dehydrogenase (*GAPDH*), and mRNA abundance was calculated using the 2^−ΔΔCT^ method.

### Construction of overexpression vector for fusion genes

A short variant of two *RNF121-FOLR2* isoforms was used in the experiment, because the long variant contained an untranslated region in the middle of the transcription sequences. After they were amplified from the patient harbouring the *RNF121-FOLR2* fusion gene, *FOLR2* and *RNF121-FOLR2* sequences were ligated into pcDNA3.1/V5-His B vectors (Invitrogen, U.S.) via one-step ligation method.^14^ For the other fusion genes, donor and acceptor gene overexpression vectors ligated in pCMV6-Entry were purchased from Origene, U.S., and then fusion gene vectors were constructed by combining the target regions of the donor and acceptor gene vectors based on pCMV6-Entry (Cosmogenetech, Korea). All vectors were confirmed by Sanger sequencing.

### Cell proliferation and migration assays

CRC cell lines, DLD-1 and SW620, were obtained from the Korean Cell Line Bank (Korea) and maintained in RPMI-1640 (Gibco, U.S.), supplemented with 10% foetal bovine serum (Gibco, U.S.), at 37 °C in a 5% CO_2_ incubator. Cells (1.5 × 10^4^) were seeded in triplicate in 24-well plates. After 24 h, adhered cells were transfected with empty, acceptor gene, or fusion gene vector for 48 and 72 h. To check cell viability, 3-(4,5-dimethylthiazol-2-yl)-2,5-diphenyltetrazolium bromide (MTT; Sigma-Aldrich, U.S.) was added to washed cells at a final concentration of 0.5 mg/mL. After 1-h incubation, absorbance was measured by spectrophotometry at 570 nm (Hewlett packard, U.S.).

After 48-h transfection of CRC cells with the appropriate vectors, migration assay was performed in triplicate using membrane filters (8 μm pore size) in disposable 96-well chemotaxis chambers (Neuro Probe; Gaithersburg, U.S.). Cells (3 × 10^3^ and 5 × 10^3^ of DLD-1 and SW620, respectively) were resuspended (50 μL), loaded into the upper chambers on membrane filters coated with 5 mg/mL fibronectin, and incubated for 4 h at room temperature. After 18 h, cells beneath the membrane were fixed, stained with Hoechst33342 (Sigma-Aldrich, U.S.), and counted by fluorescence microscopy at 10× magnification.

### Statistical analysis

Clinicopathological information was last updated in October 2017, more than 9 years after the first patient’s diagnosis. Clinicopathological features, including sex, tumour location, histological differentiation, perineural invasion, invasion depth, lymphovascular invasion, lymph node metastasis, and status of microsatellite instability, were analysed for the presence of fusion genes using the Student’s *t*-test, *χ*^2^ test, or Fisher’s exact test. Cumulative survival plots were generated using the Kaplan–Meier method, and significance was compared using the log-rank test. Statistical significance was set at *P* < 0.05. Statistical calculations were performed using IBM SSPS version 23.

## Results

### Identification and validation of fusion genes in CRCs

**Fig. 1:**
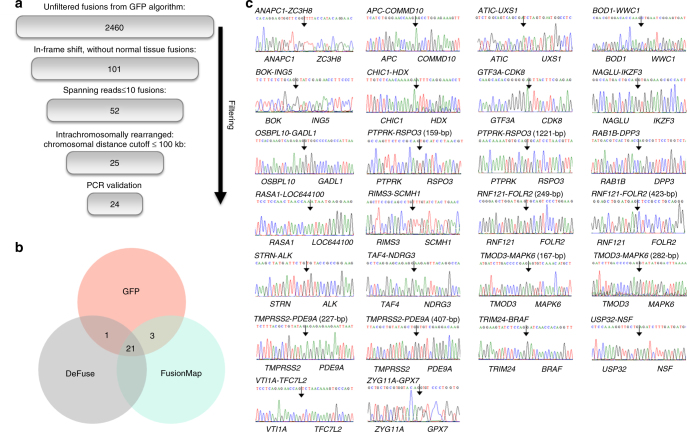
Identification and validation of fusion genes in CRC. **a** Overview of fusion gene filtering process. **b** Cross-validation with deFuse and FusionMap algorithms prior to PCR validation. GFP algorithm was used as the first detection tool, and then deFuse and FusionMap algorithms were applied to the 25 genes. **c** RT-PCR validation. Sanger sequencing confirmed 24 out of 25 fusion genes. An arrow denotes a junction point of a fusion gene

We performed RNA-seq of 147 CRC patients (147 tumour tissues and 47 matched normal tissues) in our previous study.^9^ We applied a screening process to identify significant fusion genes in CRC (Fig. 1a). Using GFP algorithm, we identified 2460 fusion genes, including 101 in-frame shifts that were not expressed in normal tissues. Next, we filtered for additional criteria, such as spanning reads ≥10 and intrachromosomal rearrangement distance cutoff ≥100 kb, and identified 25 fusion genes. FusionMap and deFuse algorithms were applied to the 25 genes for cross-validation, and all genes corresponded to two out of three algorithms (Fig. 1b). We validated 24 of 25 fusion genes via RT-PCR and Sanger sequencing (12.9%) (Fig. 1c, Table 1). We reported six of these fusion genes, including tropomyosin 3 (*TPM3*)*-NTRK1*, lamin A/C (*LMNA*)*-NTRK1*, protein tyrosine phosphatase receptor type K (*PTPRK*)*-RSPO3*, N-acetyl-alpha-glucosaminidase (*NAGLU*)*-IKZF3*, general transcription factor IIIA (*GTF3A*)*-CDK8*, and RAS p21 protein activator 1 (*RASA1*)*-LOC644100*, in our previous study.^9^ Striatin (*STRN*)*-ALK* fusion was previously reported in lung cancer, thyroid carcinomas, and colon adenocarcinoma.^15–17^ Additionally, we found *APC* and NDRG family member 3 (*NDRG3*) fusions consisting with unreported partner genes.^18, 19^ Most of the fusion genes we identified were from different individual patients (*n* = 1), except *NTRK1* (*n* = 3) and *RSPO3* fusions (*n* = 2) (Table 1).

**Table 1:** Characterisation of fusion genes determined by RNA-seq

Donor gene	Acceptor gene	In-frame shift	Count	Sample ID	Mid distance	Transciption strand	Gene 1	Break point 1	Gene 2	Break point 2	deFuse	FusionMap	High expression	Function	Reference	
															CRC	Other cancers
*ANAPC1*	*ZC3H8*	2–>0	1	13	375,316	−	*ANAPC1*	chr2: 112614191	*ZC3H8*	chr2: 112989507	O	O				
***APC***	***COMMD10***	2–>0	1	16	3,475,924	++	*APC*	chr5: 112151290	*COMMD10*	chr5: 115627214	O	O		APC; supprresor		
*ATIC*	*UXS1*	2–>0	1	4	109,465,313	+−	*UXS1*	chr2: 106717570	*ATIC*	chr2: 216182883	O	O				
*BOD1*	*WWC1*	2–>0	1	14	5,155,548	−+	*WWC1*	chr5: 167887655	*BOD1*	chr5: 173043203	O	O				
*BOK*	*ING5*	0–>1	1	6	142,177	++	*BOK*	chr2: 242501891	*ING5*	chr2: 242644068	O	O				
*CHIC1*	*HDX*	2–>0	1	19	10,812,212	+−	*CHIC1*	chrX: 72804408	*HDX*	chrX: 83616620	O	O				
					10,891,185	+−			*HDX*	chrX: 83695593^*^						
***GTF3A***	***CDK8***	2–>0	1	7	1,075,866	−−	*CDK8*	chr13: 26923209	*GTF3A*	chr13: 27999075	O	O	Donor and acceptor	CDK8; kinase	COAD (9)	
***LMNA***	***NTRK1***	0–>1	1	18	739,572	++	*LMNA*	chr1: 156105740	*NTRK1*	chr1: 156845312	O	O	Acceptor	NTRK1; kinase, oncogene	COAD (9)	Spitzoid neoplasm (COSMIC)
***NAGLU***	***IKZF3***	0–>1	1	3	2,770,478	+−	*IKZF3*	chr17: 37922746	*NAGLU*	chr17: 40693224	O	O	Donor and acceptor		COAD (9)	
*OSBPL10*	*GADL1*	2–>0	1	15	1,101,625	++	*GADL1*	chr3: 30769907	*OSBPL10*	chr3: 31871532	O	O				
***PTPRK***	***RSPO3***	0–>1	1	19	1,371,611	−+	*RSPO3*	chr6: 127469793	*PTPRK*	chr6: 128841404		O	Acceptor	PTPRK-RSPO3; oncogene	COAD, READ (20)	ESCA, STAD (20)
			1	10	1,035,784	−+			*PTPRK*	chr6: 128505577						
*RAB1B*	*DPP3*	2–>0	1	17	210,011	++	*RAB1B*	chr11: 66039928	*DPP3*	chr11: 66249939	O	O				
***RASA1***	***LOC644100***	1–>2	1	11	28,829,615	++	*RASA1*	chr5: 86564807	*LOC644100*	chr5: 115394422		O	Acceptor	RASA1; supressor	COAD (9)	
*RIMS3*	*SCMH1*	0–>1	1	14	407,181	−−	*RIMS3*	chr1: 41107381	*SCMH1*	chr1: 41514562		O				
***RNF121***	***FOLR2***	2–>0	1	9	291,744	++	*RNF121*	chr11: 71640170	*FOLR2*	chr11: 71931914	O	O	Acceptor			
					289,435	++			*FOLR2*	chr11: 71929605^*^						
***STRN***	***ALK***	0–>1	1	14	7,696,827	++	*ALK*	chr2: 29446394	*STRN*	chr2: 37143221	O	O	Acceptor	ALK; kinase, oncogene	COAD (17)	KIRP, THCA (20)
*TAF4*	*NDRG3*	2–>0	1	12	25,277,811	++	*NDRG3*	chr20: 35294795	*TAF4*	chr20: 60572606	O	O				
***TMOD3***	***MAPK6***	2–>0	1	1	183,622	++	*TMOD3*	chr15: 52155207	*MAPK6*	chr15: 52338829	O	O		MAPK6; kinase		
					183,607	++			*MAPK6*	chr15: 52338814^*^						
*TMPRSS2*	*PDE9A*	0–>1	1	5	1,303,676	−+	*TMPRSS2*	chr21: 42848504	*PDE9A*	chr21: 44152180	O	O				PRAD (20)
					1,303,376	−+			*PDE9A*	chr21: 44151880^*^						
***TPM3***	***NTRK1***	0–>1	1	2	2,701,487	−+	*TPM3*	chr1: 154142876	*NTRK1*	chr1: 156844363	O	O	Acceptor	NTRK1; kinase, oncogene	COAD (9)	SARC, THCA (20)
			1	5	2,702,436	−+			*NTRK1*	chr1: 156845312						
					2,702,996	−+			*NTRK1*	chr1: 156845872						
***TRIM24***	***BRAF***	0–>1	1	8	2,283,414	−+	*TRIM24*	chr7: 138203934	*BRAF*	chr7: 140487348	O	O		BRAF; kinase, oncogene	READ (20)	LIHC (20)
*USP32*	*NSF*	0–>1	1	14	13,631,493	−+	*NSF*	chr17: 44791351	*USP32*	chr17: 58422844	O	O				
*VTI1A*	*TCF7L2*	2–>0	1	16	614,020	++	*VTI1A*	chr10: 114286923	*TCF7L2*	chr10: 114900943	O	O			COAD (COSMIC)	
*ZYG11A*	*GPX7*	2–>0	1	13	236,289	−−	*GPX7*	chr1: 53072356	*ZYG11A*	chr1: 53308645	O	O				

Fusion genes were identified by GFP algorithm. Bold letters in first and second rows indicate finally selected fusion genes. Break points were filled in depending on Sanger sequencing results. Transcription strand column was written in the order of donor and acceptor gene (+; sense strand, −; antisense strand). The different break points of NGS and Sanger sequencing were marked with asterisks (*). According to NGS, the break points of *HDX* and *MAPK6* were located on chrX: 83724583 and chr15: 52342190, respectively, and *FOLR2* and *PDE9A* had only one break point (chr11: 71931914, chr21: 44152180, respectively). Gene function was described as kinase, oncogene, or tumour suppressor gene O; cross-validated fusion gene,

*COAD* colon adenocarcinoma, *ESCA* oesophageal carcinoma, *KIRP* kidney renal papillary cell carcinoma, *LIHC* liver hepatocellular carcinoma, *PRAD* prostate adenocarcinoma, *READ* rectal adenocarcinoma, *SARC* sarcoma, *STAD* stomach adenocarcinoma, *THCA* thyroid carcinoma

### Clinicopathological significance of CRCs positive for fusion genes

The clinicopathological features was investigated based on CRC patients positive (*n* = 19) for the 24 fusion genes. Microsatellite instability (MSI-H) associated with the presence of fusion genes (*P* = 0.025) and CRC positive for fusion genes exhibited smaller size and poor histological differentiation compared to CRC negative for fusion genes (*P* = 0.050 and *P* = 0.093, respectively). Other clinicopathological features, including sex, tumour location, perineural invasion, invasion depth, lymphovascular invasion, and lymph node metastasis, were not associated with fusion gene status of CRC samples (Table 2). CRC patients positive for fusion genes were diagnosed at diverse clinical stages (stage I = 3, stage II = 5, stage III = 11). Kaplan–Meier survival analysis showed no significant correlation between overall survival and presence of fusion genes in 147 CRC patients (fusion negative-CRC = 90.4 ± 2.70 months; fusion positive-CRC = 88.4 ± 4.46 months, *P* = 0.277) (Fig. S1).

**Table 2:** Relationship between fusion genes revealed by RNA-seq and clinicopathological characteristics in 147 patients with colorectal cancer

	(No.)	Fusion genes		*P* value
		Absent	Present	
Age (years)	147	60.1 ± 1.00	60.7 ± 2.73	0.852
Size (cm)	147	5.81 ± 0.19	4.76 ± 0.33	0.050
Sex				0.819
Male	77	68 (88.3)	9 (11.7)	
Female	70	60 (85.7)	10 (14.3)	
Location				0.162
Right colon	56	46 (82.1)	10 (17.9)	
Left colon	91	82 (90.1)	9 (9.9)	
Histological type^a^				0.093
Well	27	23 (85.2)	4 (14.8)	
Moderately	111	99 (89.2)	12 (10.8)	
Poorly + Mucinous	9	6 (66.7)	3 (33.3)	
Invasion depth^b^				0.463
T1	1	1 (100.0)	0 (0.0)	
T2	16	13 (81.3)	3 (18.8)	
T3	108	95 (88.0)	13 (12.0)	
T4	22	19 (86.4)	3 (13.6)	
Perineural invasion				0.452
Negative	89	76 (85.4)	13 (14.6)	
Positive	58	52 (89.7)	6 (10.3)	
Lymphovascular emboli				0.553
Negative	116	102 (87.9)	14 (12.1)	
Positive	31	26 (83.9)	5 (16.1)	
Lymph node				
.				0.521
Negative	72	64 (88.9)	8 (11.1)	
Positive	75	64 (85.3)	11 (14.7)	
Microsatellite status				0.025
MSS+MSI−L	126	113 (89.7)	13 (10.3)	
MSI-H	21	15 (71.4)	6 (28.6)	

^a^Between well+moderately vs. poorly+mucinous

^b^Between T1 + T2 vs. T3 + T4

### Mutually exclusive oncogenicity of fusion genes in colorectal cancers

**Fig. 2:**
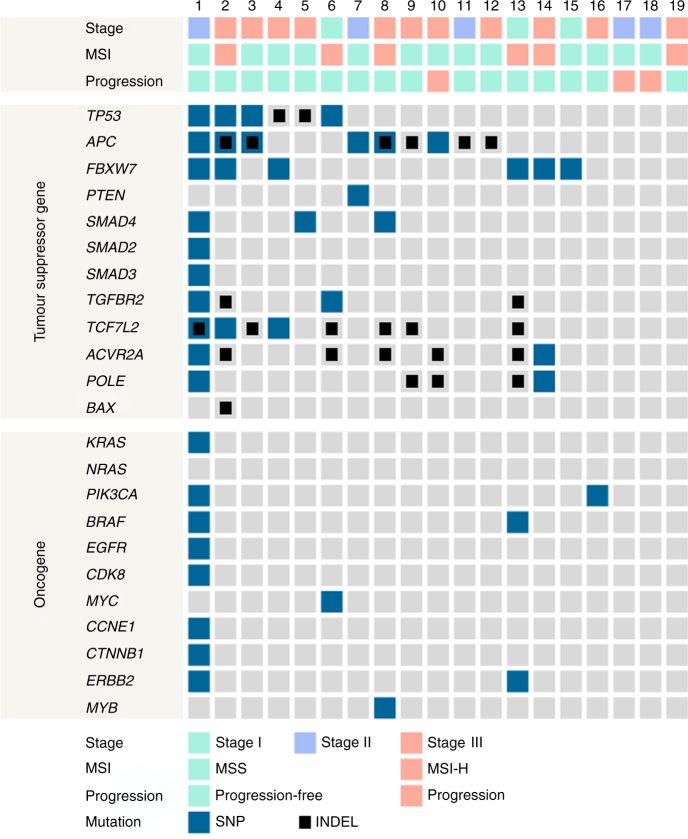
Schematic overview of mutation profiling. Mutual exclusivity of fusion genes with oncogenic mutations was represented, except *TMOD3-MAPK6*. The numbers on top designate each patient sample ID with fusion genes; 1, *TMOD3-MAPK6*; 2, *TPM3-NTRK1*; 3, *NAGLU-IKZF3*; 4, *ATIC-UXS1*; 5, *TMPRSS2-PDE9A* and *TPM3-NTRK1*; 6, *BOK-ING5*; 7, *GTF3A-CDK8*; 8, *TRIM24-BRAF*; 9, *RNF121-FOLR2*; 10, *PTPRK-RSPO3*; 11, *RASA1-LOC644100*; 12, *TAF4-NDRG3*; 13, *ANAPC1-ZC3H8* and *ZYG11A-GPX7*; 14, *BOD1-WWC1*, *RIMS3-SCMH1*, *STRN-ALK*, and *USP32-NSF*; 15, *OSBPL10-GADL1*; 16, *APC-COMMD10* and *VTI1A-TCF7L2*; 17, *RAB1B-DPP3*; 18, *LMNA-NTRK1*; 19, *CHIC1-HDX* and *PTPRK-RSPO3*

SNVs were analysed compared to the Cancer Genome Atlas (TCGA) data.^4^ To reduce the potential error of germline variants in the RNA-seq analysis, we used paired tumour–normal tissue data and dbSNP137. Fusion genes identified in this study were mutually exclusive to oncogenic mutations (Fig. 2). Somatic mutations in various tumour suppressor genes were observed in CRC patients positive for fusion genes. In contrast, in many cases, patients harbouring these fusion genes did not contain non-synonymous somatic mutations in *KRAS*, NRAS proto-oncogene, GTPase (*NRAS*), *PIK3CA*, *BRAF*, or other putative oncogenes. In addition, there were no somatic mutations in either partner genes within fusions. It was noted that one patient contained non-synonymous somatic mutations in both tumour suppressor genes and oncogenes in addition to tropomodulin 3 (*TMOD3*)*-*mitogen-activated protein kinase 6 (*MAPK6*) fusion gene, despite being microsatellite stable. Interestingly, it had three mutations in the DNA polymerase epsilon catalytic subunit (*POLE*) gene (NM_006231: exon39: c.5239G>A: p.D1747N, exon35: c.4522C>T: p.R1508C, and exon14: c.1376C>T: p.S459F), which was previously reported as a driver of hypermutated CRC,^4^ while the other patients did not have a *POLE* mutation or had only one *POLE* mutation.

### Prediction of oncogenic fusion genes in colorectal cancers

**Fig. 3:**
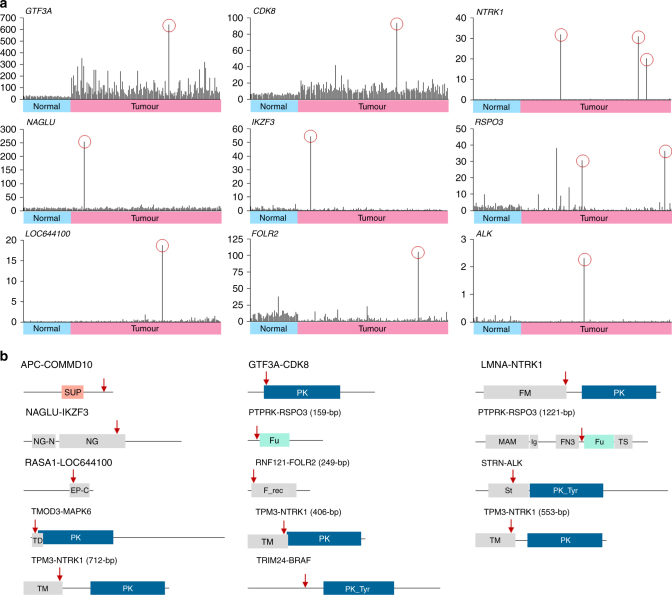
Predictions of biological functions of fusion genes. **a** Gene expression of donor or acceptor genes. Only the gene expression data of patients with high expression of donor or acceptor genes were presented. Red circle depicts patient with each fusion gene. The X-axis represents 47 paired normal samples (sky blue) and 147 tumour samples (pink). The Y-axis represents fragments per kilobase million (FPKM). **b** Schematic protein structure of fusion genes. Red arrow indicates fusion site. Protein domain abbreviations are as follows: EP-C, ARF7 effector protein C-terminus; FM, filament; FN3, fibronectin type III; F_rec, folate receptor family; Fu, furin-like repeat and cysteine-rich; Ig, immunoglobulin I-set; MAM, meprin/A5/mu; NG, NAGLU tim-barrel; NG-N, NAGLU N-terminal; PK, protein kinase; PK_Tyr, protein tyrosine kinase; St, striatin family; SUP, APC tumour suppressor protein; TD, tropomodulin; TM, tropomyosin; TS, thrombospondin type 1

Among 24 fusion genes, 11 fusions (*APC-*COMM domain containing 10 (*COMMD10*), *GTF3A-CDK8*, *LMNA-NTRK1*, *NAGLU-IKZF3*, *PTPRK-RSOP3*, *RASA1-LOC644100*, *RNF121-FOLR2*, *STRN-ALK*, *TMOD3-MAPK6*, *TPM3-NTRK1*, and tripartite motif containing 24 (*TRIM24*)*-BRAF*) from 13 patients (8.8%) were identified as oncogenic fusion genes according to their expression value, determined from outlier analysis, and function as an oncogene, tumour suppressor gene, or kinase (Fig. 3, Table 1). Expression analysis revealed that patients harbouring the fusion genes, *GTF3A-CDK8*, *LMNA-NTRK1*, *NAGLU-IKZF3*, *PTPRK-RSOP3*, *RASA1-LOC644100*, *RNF121-FOLR2*, *STRN-ALK*, or *TPM3-NTRK1*, exhibited higher expression of donor or acceptor gene compared to the other patients (Fig. 3a). Furthermore, the acceptor genes of *GTF3A-CDK8*, *LMNA-NTRK1*, *PTPRK-RSOP3*, *STRN-ALK*, *TMOD3-MAPK6*, *TPM3-NTRK1*, and *TRIM24-BRAF* were characterised to have kinase functional domains and oncogenicity (Fig. 3b). Functional domain analysis of *APC-COMMD10* and *RASA1-LOC644100* fusion genes showed truncation of APC (293 aa/325 aa) and RASA1 (179 aa/253 aa), respectively, which may induce loss of function of each tumour suppressor (Fig. S2).

### Functional validation of CDK8, IKZF3, FOLR2, and ALK fusion genes in colorectal cancers

We selected *GTF3A-CDK8*, *LMNA-NTRK1*, *NAGLU-IKZF3*, *RNF121-FOLR2*, *STRN-ALK*, and *TPM3-NTRK1* for functional analysis, according to their expression values. However, we recently investigated the functional roles of *LMNA-NTRK1* and *TPM3-NTRK1* in CRC^9^ and the oncogenic role of *PTPRK-RSOP3* was previously reported.^7^ Therefore, we performed in vitro functional analysis of only four fusion genes, *GTF3A-CDK8*, *NAGLU-IKZF3*, *RNF121-FOLR2*, and *STRN-ALK*, in the present study. First, RT-PCR was conducted to detect the presence of the fusion genes in various CRC cell lines, including DLD-1, HT-29, SW480, SW620, and HCT15. The short variant of *RNF121-FOLR2* fusion gene was confirmed present in HT-29 cells, whereas no other fusion genes were detected in the cell lines (Fig. S3A). To accurately validate this, the full sequence of *RNF121-FOLR2* was amplified in HT-29 cells and the sequencing result aligned with reference sequences (Fig. S3B). However, *RNF121-FOLR2* expression in HT-29 cells was significantly lower than that in the CRC patient, which was not suitable for knockdown experiments (Fig. S3C).

**Fig. 4:**
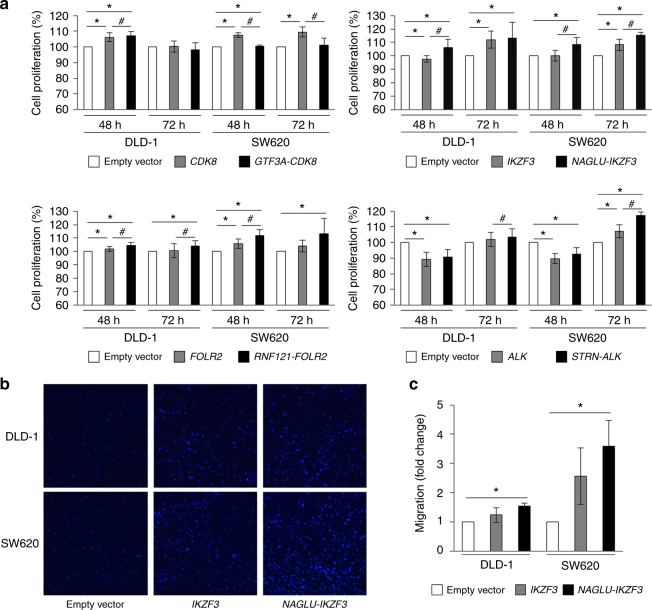
Biological function test. **a** Cell proliferation in CRC cells. Each MTT assay was performed three times. Empty vector is pCMV6-myc-DDK, except *RNF121-FOLR2*, which used pcDNA3.1-V5-His B. *P < 0.05 compared to empty vector, determined by the Student’s *t*-test; ^#^*P* < 0.05 compared to each acceptor gene expression vector, determined by the Student’s *t*-test. **b** Cell migration assay of *NAGLU-IKZF3* was performed three times. Harvested cells were seeded in transwell 48 h after transfection. *P < 0.05 compared to empty vector, determined by the Student’s *t*-test

Next, we performed MTT assay to determine the effect of the fusion genes on cell proliferation (Fig. 4a). No significant change was observed in cells overexpressing *GTF3A-CDK8*. Cell proliferation of CRC cell lines overexpressing *NAGLU-IKZF3* and *RNF121-FOLR2* increased at both 48 and 72 h after transfection, compared to the negative control. In addition, CRC cells overexpressing *STRN-ALK* grew rapidly at 72 h, compared to the negative control. To investigate the effect of the fusion genes on migration capacity of CRC cells, we performed migration assay 48 h after transfection of overexpression vectors. CRC cells overexpressing *NAGLU-IKZF3* exhibited increased migration capacity, compared to cells transfected with empty or *IKZF3* vector (Fig. 4b, c). Especially, migration of SW620 cells overexpressing *NAGLU-IKZF3* increased by 3.5-fold (Fig. 4b, c). However, CRC cells transfected with *GTF3A-CDK8*, *RNF121-FOLR2*, or *STRN-ALK* did not show aberrant migration (data not shown). Additionally, we conducted cell cycle analysis via propidium iodide staining, but no apoptotic effect was observed by the fusion genes (Fig. S4).

Therefore, our results demonstrated that expression of *NAGLU-IKZF3*, *RNF121-FOLR2*, and *STRN-ALK* enhanced cell proliferation, while expression of *NAGLU-IKZF3* promoted cell migration.

## Discussion

We identified 24 fusion genes, including novel fusion genes that were not previously reported, through RNA-seq of 147 CRC patients. Among those, we determined that 11 fusion genes were oncogenic according to gene expression and function. Finally, we validated in vitro the oncogenic functions of *CDK8*, *IKZF3*, *FOLR2*, and *ALK* fusion genes in CRC cell lines.

Several studies identified various fusion genes in CRC, including *NAV2-TCF7L1*, *RSPO*, *BRAF*, *NTRK3*, *RET*, and *RAS* fusion genes, as good candidates of molecularly targeted therapy.^4, 7, 8^ Our strict filtering approach uncovered novel fusion genes in CRC, compared to fusion gene databases, such as Tumour Fusion Gene Data Portal^20^ and Catalogue of Somatic Mutations in Cancer (COSMIC), and other studies. We reported 14 novel fusions out of the 24 fusion genes identified, including anaphase promoting complex subunit 1 (*ANAPC1*)*-*zinc finger CCCH-type containing 8 (*ZC3H8*), *APC-COMMD10*, 5-aminoimidazole-4-carboxamide ribonucleotide formyltransferase (*ATIC*)*-*UDP-glucuronate decarboxylase 1 (*UXS1*), biorientation of chromosomes in cell division 1 (*BOD1*)*-*WW and C2 domain containing 1 (*WWC1*), Bcl2 family apoptosis regulator (*BOK*)*-*inhibitor of growth family member 5 (*ING5*), cysteine rich hydrophobic domain 1 (*CHIC1*)*-*highly divergent homeobox (*HDX*), oxysterol binding protein like 10 (*OSBPL10*)*-*glutamate decarboxylase like 1 (*GADL1*), Ras-related protein Rab-1B (*RAB1B*)*-*dipeptidyl peptidase 3 (*DPP3*), regulating synaptic membrane exocytosis 3 (*RIMS3*)*-*Scm polycomb group protein homologue 1 (*SCMH1*), *RNF121-FOLR2*, TATA-box binding protein associated factor 4 (*TAF4*)*-NDRG3*, *TMOD3-MAPK6*, ubiquitin specific peptidase 32 (*USP32*)*-*N-ethylmaleimide sensitive factor (*NSF*), and zyg-11 family member A (*ZYG11A*)*-*glutathione peroxidase 7 (*GPX7*). *MAPK6-TMOD3*, with different direction and junction point from *TMOD3-MAPK6*, was identified by Hu et al.^20^ In our study, we confirmed expression of *RNF121-FOLR2* in HT-29 cells, which was contrary to Nome et al. who identified expression of fusion genes via RNA-seq of various CRC cell lines, including HT-29.^21^ The discrepancy may be due to the low expression of *RNF121-FORL2* in HT-29 cells, which may result in significantly fewer mapping reads by RNA-seq. *RNF121-FOLR2* expression in the CRC patient was 10 times more abundant than in HT-29 cells (Fig. S3C).

Clinicopathological analysis determined that MSI status, more specifically MSI-H, of CRC patients positive for fusion genes correlated with the presence of fusion genes. The association of the presence of fusion genes and MSI status is unclear; fusion genes are suggested to be upregulated in MSI tumours. Kloosterman et al. reported that gene fusion tended to occur in CRC patients with MSI-H (*P* = 0.007),^8^ and Kalvala et al. reported that 55% of CRC patients with MSI tended to have fusion genes (*n* = 54; *P* = 0.166).^22^ Continuous studies are required to clarify the relation between the presence of fusion genes and MSI. Histotype-genotype associations based on fusion genes have been extensively investigated and some reports suggest that harbouring a fusion gene was significantly associated with certain histological types of cancer.^23–26^ In the present study, correlation analysis regarding histological types revealed that CRC patients harbouring fusion genes tended to display poor histological differentiation compared to CRC negative for fusion genes (*P* = 0.093). However, more cases are necessary to be reported for statistical relevance, since this result only reveals trends.

We implicated the oncogenic potential of these fusion genes because our SNV profiling determined that the presence of fusion genes was mutually exclusive with the presence of oncogenes in CRC patients, exception of one patient expressing *TMOD3-MAPK6* (*P* = 0.029), concurrent with previous reports wherein tumour samples harbouring fusion genes tended to have significantly fewer oncogene mutations.^27, 28^ However, cancer progression of the patient with *TMOD3-MAPK6* may be affected by oncogene and tumour suppressor gene mutations involving *POLE*, in addition to the fusion gene.^4^

We selected 11 fusion genes (*APC- COMMD10, GTF3A-CDK8*, *LMNA-NTRK1*, *NAGLU-IKZF3*, *PTPRK-RSOP3*, *RASA1-LOC644100*, *RNF121-FOLR2*, *STRN-ALK*, *TMOD3-MAPK6*, *TPM3-NTRK1*, and *TRIM24-BRAF*) that were predicted as stronger oncogenes due to gene expression or function. Interestingly, we discovered fusion genes that comprised of tumour suppressor genes, *APC* and *RASA1*. Functional domain analysis showed truncation of *APC* in *APC*-*COMMD10* (1–293 aa in 325 aa) and *RASA1* in *RASA1*-*LOC644100* (1–179 aa in 253 aa). Truncated APC forms, especially those lacking the C-terminal, are commonly expressed in CRC, while wild type APC is expressed in normal tissue.^29, 30^ The beta-catenin binding region (1020 aa-1638 aa) of APC is important for inhibiting Wnt signalling activation via cytosolic beta-catenin turnover.^29, 31–33^ Tighe et al. reported that truncated APC without beta-catenin-binding region initiated chromosomal instability, thereby exerting oncogenic effects.^34^ Therefore, truncated *APC* in *APC*-*COMMD10* could not have a tumour suppressor-like function because *APC*-*COMMD10* does not have any functional domain. In addition, 120RasGAP coded by *RASA1* converts active Ras-GTP to inactive Ras-GDP, which then inhibits RAS oncogene.^35, 36^ At the molecular level, Src homology 2 (SH2), Src homology 3 (SH3), pleckstrin homology (PH), and calcium-dependent phospholipid-binding (C2) domains regulate cell proliferation, migration, and apoptosis in accordance with their sub-binding partners.^37–41^ Tumour suppressor activity may be obliterated in *RASA1*-*LOC644100*, since the fused region of *RASA1* does not involve all pivotal domains for inhibiting tumourigenesis.^42^ Therefore, its truncation in the fusion gene may promote tumourigenic mechanisms in CRC. These hypotheses were partially supported by the lack of somatic mutations of *APC* and *RASA1* identified in our cohort of CRC samples that expressed *APC* and *RASA1* fusion genes.

There are several discrepancies regarding the frequency and type of fusion genes in CRC between different studies. In our study, we identified 11 oncogenic fusion genes in 8.8% of our cohort. In comparison, Kloosterman et al. reported that only 2.5% of CRC expressed fusion genes, including ArfGAP with GTPase domain, ankyrin repeat, and PH domain 3 (*AGAP3*)*-BRAF*, *TRIM24-BRAF*, discs large MAGUK scaffold protein 1 (*DLG1*)*-BRAF*, echinoderm microtubule associated protein like 4 (*EML4*)*-NTRK3*, ribosome binding protein 1 (*RRBP1*)*-RET*, *USP9X-*embryonic stem cell-expressed Ras (*ERAS*), and eukaryotic translation initiation factor 3 subunit E (*EIF3E*)*-RSOP2* fusion genes.^8^ These discrepancies may be due to differences in sample collection and preparation and bioinformatics analysis pipeline. Furthermore, the functional significance of fusion genes in CRC varies between studies. Seshagiri et al. reported recurrent fusion genes involving R-spondin family members, *RSOP2* and *RSPO3*, in 10% of colon cancers.^7^ However, Kloosterman et al., Shinmura et al., and our present study demonstrated that *RSPO* fusion genes were lowly expressed in colon cancers (0.35%, 4%, and 1.36%, respectively).^8, 43^ This may be due to sampling bias, bioinformatics tools, or selection bias, among other possible reasons. However, Kloosterman et al. suggested that the use of different bioinformatics tools between studies was not a major confounding factor.^8^ Additional studies are necessary to investigate these discrepancies further.

In the present study, we performed functional analysis of four fusion genes, *GTF3A-CDK8*, *NAGLU-IKZF3*, *RNF121-FOLR2*, and *STRN-ALK*. We validated the biological functions of the fusion genes in vitro. In this study, although *CDK8* expressed intact protein kinase domain, overexpression of *GTF3A-CDK8* did not have any effect on cell proliferation, contrary to our *CDK8* overexpression data and a previous study in which *CDK8* activated the Wnt/beta-catenin signalling pathway in CRC.^44^ The CDK8 kinase module, comprised of mediator 12, mediator 13, cyclin C, and CDK8, binds beta-catenin, which promotes the transcription of oncogenes.^45^ The αB helix (3 aa-12 aa) at the N-terminal of CDK8 is important for recognition of cyclin C,^46^ and the CDK8/cyclin C complex recruits the remaining components of the CDK8 kinase module, mediator 12 and 13.^47^ In CRC overexpressing *GTF3A-CDK8*, the CDK8 module may not properly form due to the absence of the region that interacts with cyclin C in the fusion gene. Thus, we suggest that the N-terminal of CDK8 may play a pivotal role in oncogenesis in CRC.

Overexpression of *NAGUL-IKZF3* significantly increased cell growth and migration, compared to cells overexpressing only *IKZF3*. *IKZF3* expression was previously demonstrated to mediate cancer metastasis by promoting anchorage independence,^48^ and cell proliferation of a breast cancer cell line possessing VAMP-associated protein B and C (*VAPB*)*-IKZF3* was suppressed when *IKZF3* was knocked down.^49^ We demonstrated that *NAGUL-IKZF3* expression may influence both tumourigenesis and metastasis in CRC.

CRC cells overexpressing *RNF121-FOLR2* exhibited increased cell proliferation. Folate receptor beta, coded by *FOLR2*, was reported to localise to activated macrophages that amass in tumours and at inflammation sites.^50–52^ Folate receptor beta was expressed in tumour-associated macrophages (TAMs) that caused immune tolerance, enhancing angiogenesis, increasing tumour cell migratory and invasive abilities, and reducing cell apoptosis and sensitivity to anticancer drugs.^53–55^ It is unclear whether *FOLR2* expression in tumour cells recruits TAMs or TAMs highly express *FOLR2*. Regardless, immunotoxins targeting folate receptor beta reduced tumour growth in glioma xenograft models.^50^ Hence, the presence of *RNF121-FOLR2* may be a potential prognostic marker for folate-mediated, anti-inflammatory drugs.

Overexpression of *STRN-ALK* increased CRC cell proliferation. Cells expressing *STRN-ALK* increased thyroid-stimulating, hormone-independent cell proliferation and developed tumours in nude mice.^16^ The ALK inhibitor, crizotinib, was approved by the US Food and Drug Administration as a chemotherapeutic treatment for lung cancer patients positive for *EML4-ALK*.^56^ Although the function of *STRN-ALK* related with CRC was not studied in vitro, Yakirevich et al. conducted a clinical trial of the ALK inhibitor, ceritinib, which was 20-fold more effective than crizotinib, on a cohort that included a CRC patient positive for *STRN-ALK* in a pre-clinical setting.^17^ Ceritinib treatment decreased tumour size of the patient with *STRN-ALK*, but resistance to ceritinib developed after 9 months.^17^ Therefore, we also suggest that ALK inhibitors may be provided to patients positive for *STRN-ALK* as an initial drug.

In conclusion, we comprehensively analysed fusion genes via various fusion algorithms, clinicopathological information, and SNV data in CRC patients, identified the significant fusion genes, and investigated their function. We demonstrated the oncogenic capacity of several fusion genes detected in this study through analysis of SNVs, gene expression, and gene function, and we determined that *NAGLU-IKZF3*, *RNF121-FOLR2*, and *STRN-ALK* had tumourigenic effects in CRC. Therefore, these fusion genes may be good candidates as molecular targets for the development of cancer therapy in precision medicine.

## Electronic supplementary material

Supplementary figure 1

Supplementary figure 2

Supplementary figure 3

Supplementary figure 4

Supplementary table 1

Supplementary table 2

Supplementary Information
