# Supplementary figures and images for: Integrative analysis of oncogenic fusion genes and their functional impact in colorectal cancer
Source: Br J Cancer. 2018 Jun 29;119(2):230–40. doi: 10.1038/s41416-018-0153-3 (PMC6048111; doi:10.1038/s41416-018-0153-3)

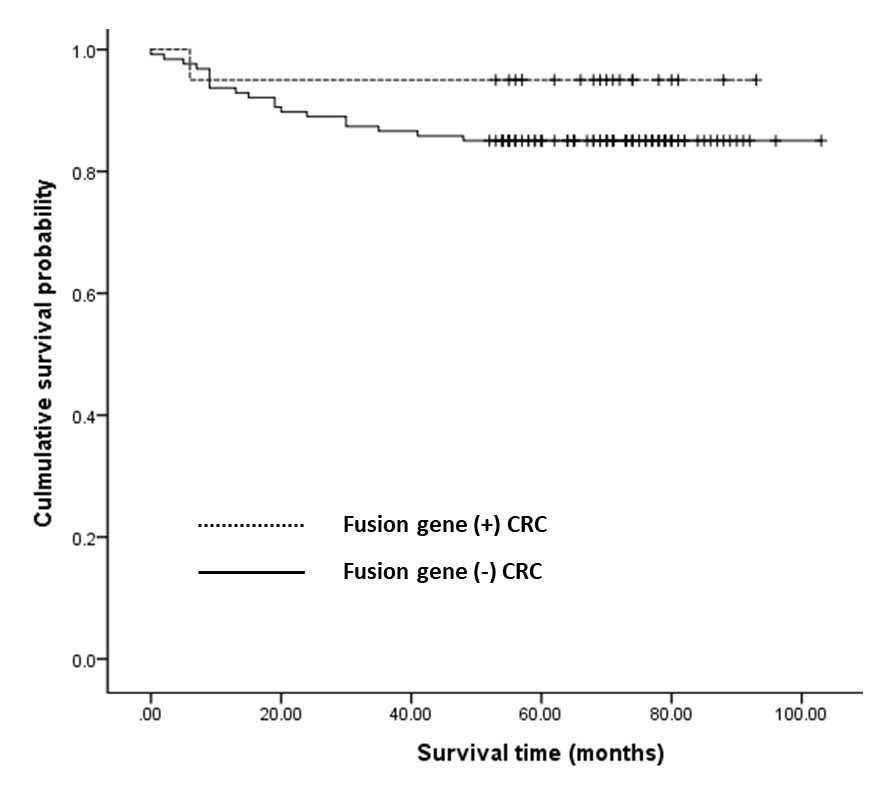

Supplement: Supplementary file 1 — Supplementary figure 1 [file 41416_2018_153_MOESM1_ESM.tif]

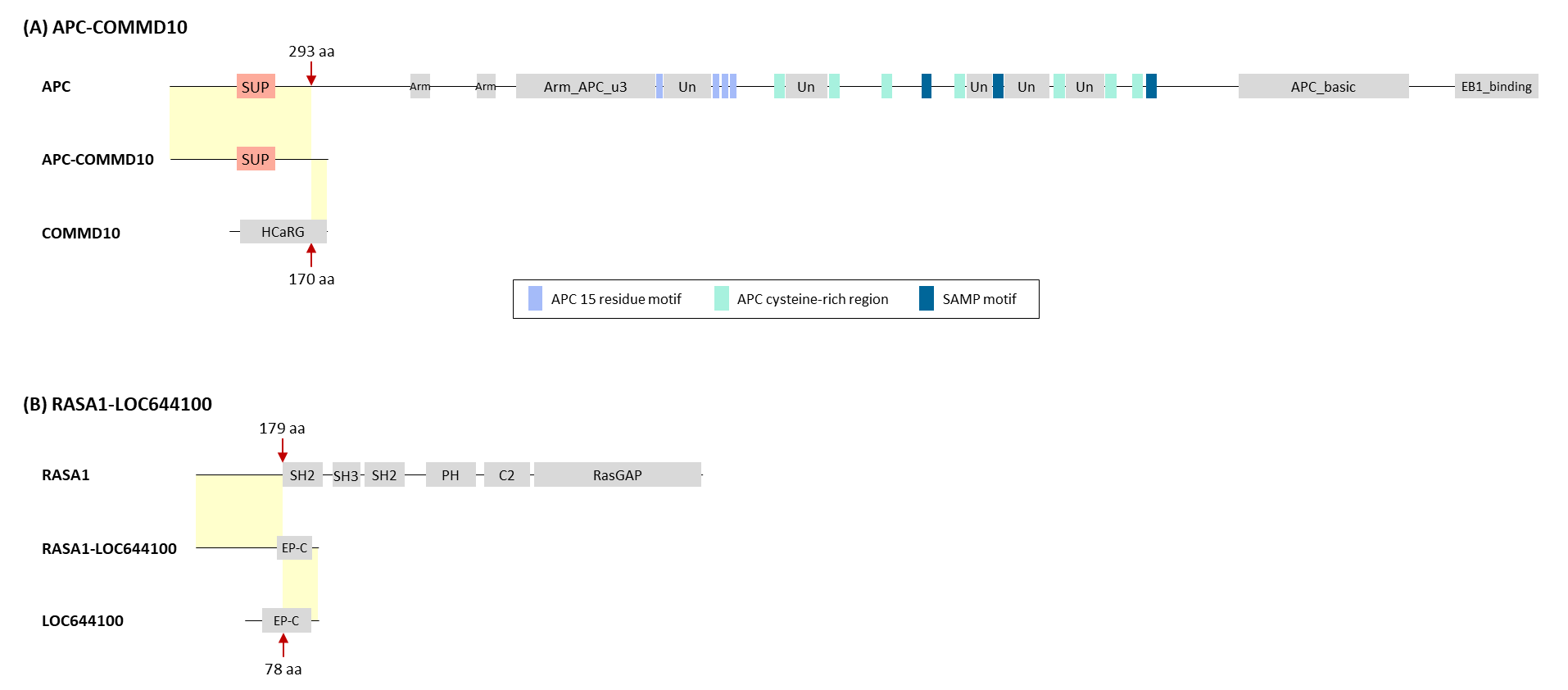

Supplement: Supplementary file 2 — Supplementary figure 2 [file 41416_2018_153_MOESM2_ESM.tif]

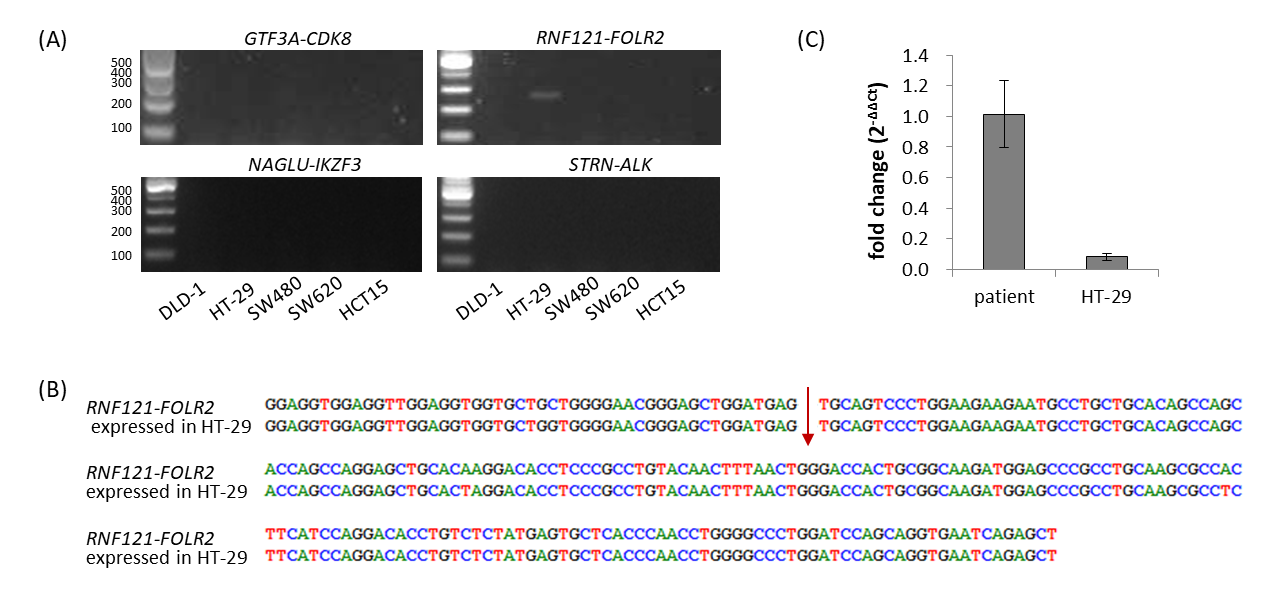

Supplement: Supplementary file 3 — Supplementary figure 3 [file 41416_2018_153_MOESM3_ESM.tif]

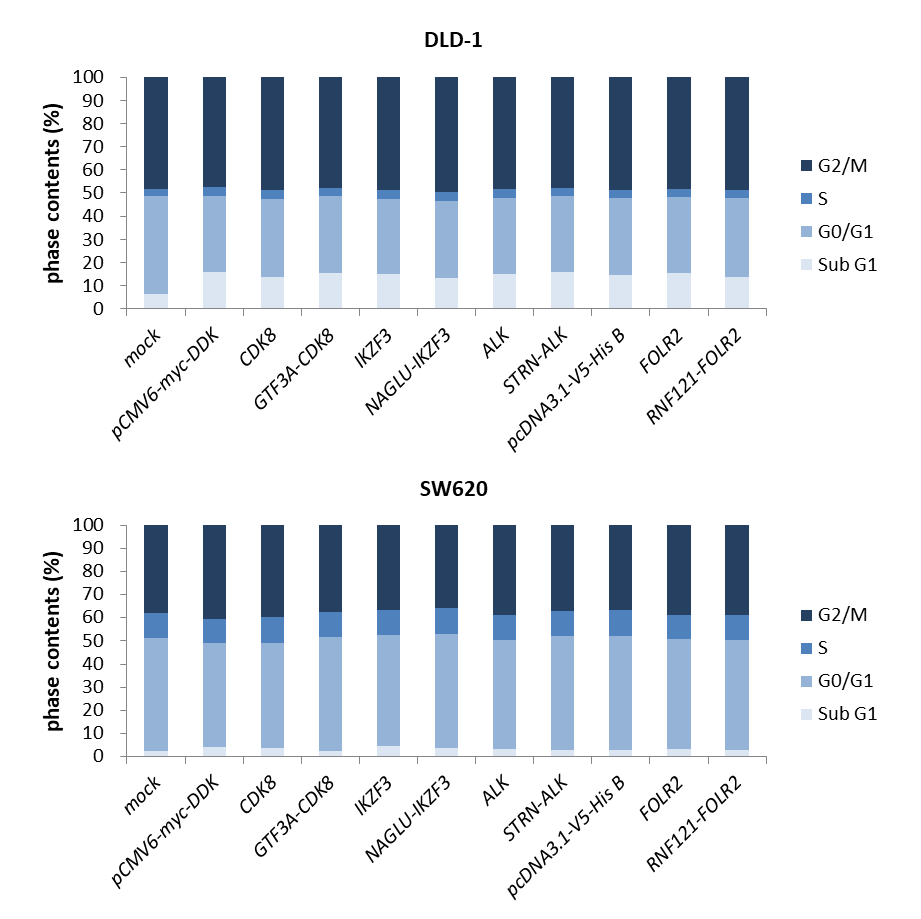

Supplement: Supplementary file 4 — Supplementary figure 4 [file 41416_2018_153_MOESM4_ESM.tif]
