# Supplementary material for: Integrative analysis of oncogenic fusion genes and their functional impact in colorectal cancer
Source: Br J Cancer. 2018 Jun 29;119(2):230–40. doi: 10.1038/s41416-018-0153-3 (PMC6048111; doi:10.1038/s41416-018-0153-3)
Supplement: Supplementary file 7 — Supplementary Information [file 41416_2018_153_MOESM7_ESM.docx]

**Supplementary Figure 1.** Kaplan-Meier survival analysis of 19 patients with 24 fusion genes and 128 patients without them (fusion-positive CRC, 88.4 ± 4.46 months; fusion negative-CRC, 90.4 ± 2.70 months; *P* = 0.277)

**Supplementary Figure 2.** Schematic protein structures of APC-COMMD10 and RASA1-LOC644100. Red arrow indicates break point. APC_basic, APC basic domain; Arm, Armadillo/beta-catenin-like repeat; Arm_APC_u3, Armadiollo-associated region on APC; C2, Calcium-dependent phospholipid binding domain; EB1_binding, EB-1 binding domain; EP-C, ARF7 effector protein C-terminus; HCaRG; hypertension-related and calcium-regulated gene protein; PH, Pleckstrin homology domain; RasGAP, GTPase-activator protein for Ras-like GTPase; Un, unstructured region; SH2, Src homology 2 domain; SH3, Src homology 3 domain; SUP, APC tumour suppressor protein.

**Supplementary Figure 3.** (A) RT-PCR in 5 CRC cell lines. (B) Sanger sequencing result of *RNF121-FOLR2* expressed in HT-29 cells. Red arrow represents break point. (C) Comparison of *RNF121-FOLR2* expression between patient positive for *RNF121-FOLR2* and HT-29 cells by qRT-PCR.

**Supplementary Figure 4.** Cell cycle analysis by propidium iodide staining to examine apoptotic effect of fusion genes.

**Supplementary Table 1. Primer information for fusion gene validation.** F, forward; R, reverse.

**Supplementary Table 2. Primer information for acceptor gene expression.** F, forward; R, reverse
